# Cameroonian Physiotherapists’ Practice, Confidence, and Perception of Health Promotion for People at Risk or with Cardiovascular Diseases: A Qualitative Study

**DOI:** 10.3390/healthcare13101172

**Published:** 2025-05-17

**Authors:** Etienne Ngeh Ngeh, Rachel Young, Christopher Kuaban, Sionnadh McLean, Ben W. Strafford, Joanne Lidster

**Affiliations:** 1 Research Organization for Health Education and Rehabilitation-Cameroon (ROHER-CAM), Mankon, Bamenda P.O. Box 818, Cameroon; ckuaban@yahoo.fr; 2 Department of Allied Health Professions, Sheffield Hallam University, L108, 36 Collegiate Crescent, Sheffield S10 2BP, UK; r.young@shu.ac.uk (R.Y.); j.lidster@shu.ac.uk (J.L.); 3 Faculty of Medicine and Biomedical Sciences, University of Yaoundé I, Yaoundé P.O. Box 4021, Cameroon; 4 School of Allied Health Sciences, Charles Darwin University, Darwin, NT 0810, Australia; sionnadh.mclean@cdu.edu.au; 5 School of Sport and Physical Activity, Collegiate Hall, Collegiate Crescent, Sheffield S10 2BP, UK; b.strafford@shu.ac.uk

**Keywords:** physiotherapy, health promotion, cardiovascular diseases, risk factors, Cameroon

## Abstract

**Background:** Cardiovascular diseases (CVDs) and their risk factors are increasing with associated disability and mortality burden globally, especially in low- and middle-income countries, including Cameroon. Physiotherapist-led health promotion (PLHP) interventions provide opportunities to improve health and reduce this burden. Understanding physiotherapists’ practice, confidence, and perception is crucial for designing effective, context-specific PLHP interventions. **Methods:** This qualitative study explored physiotherapists’ practice, perceptions, and confidence in delivering PLHP to pwCVDs in Cameroon. **Results:** Sixteen participants completed the interviews, and analyses of the transcripts generated three main themes, which included (1) the perception of physiotherapists’ roles in health promotion (HP), (2) current practice of PLHP, and (3) competence in the delivery of PLHP. Physiotherapists believe that delivering HP interventions in practice is within their professional role. Current HP practice was limited to exercise, physical activity, and dietary/nutritional interventions. Participants reported a lack of knowledge and formal training in PLHP delivery. **Conclusions:** Despite intense interest in HP, HP practice among physiotherapists is limited in scope, is under-resourced, and is limited by a lack of confidence in delivering behavioural change interventions. These findings are relevant for the design of appropriate clinical training and policies for the care of pwCVDs.

## 1. Introduction

The global burden of non-communicable diseases (NCDs) has resulted in increasing numbers of people at risk or with cardiovascular diseases (pwCVDs) [1,2,3]. NCDs have emerged as a major public and clinical problem globally, particularly in Cameroon, with an increase in the annual mortality burden from 27.4% in 2000 to 37.7% in 2019 [4,5]. The prevalence of hypertension ranges from 19.8% in rural areas [6] to 47.5% in urban milieus, with a national average of 31.0% [7]. Hypertension accounts for 41.3–54.5% of diagnosed CVD cases in Cameroon [8,9]. The incidence of stroke is increasing (2.5% in 1999–2000 to 13.1% in 2011–2012), with case fatality increasing from 14.4% to 22.4% over the same decade [10]. In one hospital-based study in Cameroon, CVD accounted for 10–16% of all hospital admissions, with heart failure (38.5%) and stroke (33.3%) making up the bulk of these admissions [8]. In 2012, 12% of Cameroonian deaths were attributed to CVD [11]. The prevalence of diabetes [12], hypercholesteremia [13], tobacco use, and overweight/obesity [14] are high relative to regional and global figures [13]. It is necessary to curb the growing prevalence of pwCVDs, premature deaths, long-term disabilities, hospitalizations, and rehabilitation costs [15,16,17], and to reduce the growing burden on healthcare systems [18]. This has significant implications for practising physiotherapists in Cameroon.

The primary roles of physiotherapists are to manage/treat diseases, promote health, and to prevent disability in individuals and the general population [19,20]. Physiotherapists use health education, direct interventions, research, advocacy, and collaborative consultations to fulfil these roles [21]. In some countries, physiotherapists are expected to advocate for primary health principles, including diet, rest, exercise, weight control, tobacco use cessation, immunizations, avoidance of infectious and contagious diseases, skin care, dental hygiene, and sanitation [22].

It is well established that NCDs are associated with an unhealthy lifestyle, suggesting that lifestyle changes could reduce NCD burdens significantly [1,2]. HP programmes targeting lifestyle changes have been effective at reducing blood pressure, increasing physical activity uptake, and improving health behaviours in various populations [23,24,25,26]. These HP programmes typically include counselling, behavioural change advice, and specialist referrals for personalized care within healthcare settings [24]. A crucial aspect of effective HP is to Make Every Contact Count (MECC), where healthcare professionals use each patient interaction as an opportunity to encourage positive changes in health behaviour [27].

The Physiotherapy Summit on Global Health highlighted the need for physiotherapists to collaborate with other health practitioners to initiate and support interventions to combat NCDs using their unique skills and knowledge [22,28,29]. Given the prevalence of pwCVDs in Cameroon, strengthening Physiotherapist-Led Health Promotion (PLHP) is crucial for addressing the rising NCD burden [30]. With their frequent and extended patient interactions, physiotherapists are well-positioned to screen for and influence lifestyle behaviours that contribute to these conditions [31]. An essential part of the PLHP strategy is health education [32]. It is essential for physiotherapists to possess the following four clinical communication competencies: know (has acquired theoretical knowledge and skills), know-how (knows how to apply these skills), show-how (can competently demonstrate these skills on specific occasions), and do (can competently demonstrate these skills daily) [33,34]. Acquiring skills and competencies in a wide range of HP activities is essential for physiotherapists to feel equipped to implement PLHP for pwCVDs [34].

PLHP faces challenges across various healthcare systems due to a focus on acute care in hospitals and a need for greater competence [35,36]. Barriers such as inadequate HP training, a lack of confidence [37], skill gaps, time constraints, beliefs, reimbursement issues, and current workloads also hinder effective PLHP [38,39]. Despite the growing need for PLHP in Cameroon, it is unclear how physiotherapists are involved in HP clinically, or in policy or research. Therefore, the aim of this study was to explore physiotherapists’ practice and perceptions of their role and confidence in delivering PLHP to pwCVDs in Cameroon. The findings of this study will contribute to the global health literature on HP, particularly in low- and medium-income countries.

## 2. Materials and Methods

### 2.1. Design

This study employed a qualitative design drawing from the constructivist paradigm [40]. Constructivism operates from the position of multiple realities shaped by context and experience [40]. The constructivist paradigm urges researchers to cultivate an empathic understanding by viewing the world from the participants’ perspectives [41]. This paradigmatic approach was instrumental in guiding data collection methods, concentrating on physiotherapists’ views regarding their practice and role in HP. The Standards for Reporting Qualitative Research were adopted for the conduct and reporting of this study [42].

### 2.2. Study Area

This study was conducted among physiotherapists practicing in Cameroon [43]. Physiotherapists work in various settings, including private clinics/cabinets, public and private (religious) hospitals, military hospitals, and sports clubs, and in teaching in higher education institutions. These physiotherapists prescribe physical activity and exercise as well as advice on several components of HP to manage pwCVDs [44]. Most physiotherapists are concentrated in urban areas, with limited physiotherapy services delivered in rural areas [45].

### 2.3. Participants

Sixteen physiotherapists were purposively recruited from the sample that participated in our previous study (45). Inclusion criteria were as follows: (i) physiotherapists practising in Cameroon for at least one year, (ii) physiotherapists with at least two years of physiotherapy training, and (iii) physiotherapists providing consent to participate. Forty-three physiotherapists provided their preliminary consent to participate in the interview during an online survey [44]. Sixteen of these were purposefully recruited based on age, gender, their highest level of training, work experience, and location of the practice. This approach was used to achieve a cross-section of the sample that could offer deep insight based on their practice as physiotherapists [46]. Written informed consent was obtained from all participants prior to participating in interviews.

### 2.4. Interview Procedure and Data Collection

In accordance with an interpretive paradigm, a semi-structured interview guide was developed, with open-ended questions to explore participants’ current practice, perspectives of their role, and confidence in delivering PLHP to pwCVDs in Cameroon (Supplementary File S1). Permission to record the interview was obtained from all consenting participants to ensure the accuracy of the resulting transcripts [47]. The investigator used general familiarization questions specific to the physiotherapy practice to build rapport in the presence of the audio recording device before addressing all central questions [48]. Subsequently, the discussion moved on to their practice, confidence, and perceptions of their role in PLHP delivery. Interview recordings were transcribed, including verbal expressions and body language that reveal considerable emotions or feelings on specific issues [49]. A field diary based on comments and explanations of description and interpretation of context-specific slang or jargon was kept. All interviews were conducted face-to-face by the lead author (E.N.N.), mainly in the clinical settings of the participants between 5 February and 1 March 2024. Interviews lasted between 18 and 60 min, with a mean duration of 34 min. All interviews were audio-recorded and transcribed. We concluded that data saturation had been achieved by comparing interview data throughout the analytical process and observing that no new aspects, dimensions, or nuances of codes emerged [50].

### 2.5. Data Analysis

Thematic content analysis was condugcted using NVIVO version 15 [51,52]. A six-step thematic approach was used in the first stage, as recommended by Braun and Clarke [53]. The results of the early interviews were used to develop common categories and subcategories, and some previously defined categories were clarified with further analysis. Each category was supported with a quote from an interview transcript. The authors (E.N.N. and B.S.) compared each new topic with previous transcripts whenever it was brought up. As a result, the general categorization was able to evolve until the end of the analysis (data saturation) [53]. Following the individual analysis of each interview, two researchers (E.N.N. and B.S.) conducted a joint analysis. E.N.N. is a physiotherapist and lecturer, and B.S. is a qualitative researcher in sports and exercise science. The researchers were cognizant of their own diverse personal and professional viewpoints and experiences, some of which were considered to result from having insider status within the physiotherapy community. Although these opinions and experiences could have impacted the analysis [54], the authors were aware of the need to set aside their views and focus on the exploration of the study’s aims. Any discrepancies or additional ideas were discussed until agreement was reached and/or they were included in the findings. Two experienced qualitative (R.Y. and J.L.) and quantitative (S.M. and C.K.) co-investigators reviewed, and they refined the final findings. The experienced researchers confirmed that the analysis was credible and that themes were rooted in the data. A qualitative research report was prepared using guidelines for reporting qualitative findings [42].

## 3. Results

### 3.1. Participants Characteristics

A total of 16 physiotherapists, aged between 30 and 63 years, participated in the semi-structured interviews. Table 1 presents detailed characteristics of the study participants.

### 3.2. Qualitative Findings

Analyses of the transcripts generated 23 lower-order themes, seven higher-order themes, and three main themes, which included (1) the perception of physiotherapists’ role in HP, (2) the current practice of PLHP, and (3) confidence in the delivery of PLHP, as presented in Table 2. Complete results can be found in the supplementary file, Table S1.

#### 3.2.1. Theme 1: Perceptions of Their Roles in Health Promotion

All participants perceived HP to be well within the professional scope of physiotherapy practice. Some took the perspective that patients are less likely to know about their condition, and it is the fundamental and integral duty of physiotherapists to educate them.

*“As a physiotherapist, you go a long way to give more knowledge to the patients because most of the patients that come in … have less information concerning their conditions”*.

*P7*

A few (2/16) physiotherapists in the private sector embrace HP practice to provide a more comprehensive and attractive healthcare package for their patients. They believed that HP advice improves health outcomes, therefore providing a higher-quality service for their clients. Hence, HP is adopted and practiced in the private sector for business sustainability and the reputation of physiotherapy practice.

*“It is important because the health promotion goes with the physiotherapy. You offer it because it’s going to help people get more ... especially in the private practice, you need them to come back… to refer people… your service is better than the regular service anywhere else”.*

*P6*

Most of the participants perceived HP to be very valuable in the management and prevention of diseases, including primary prevention (P13), secondary prevention (P6) and tertiary prevention (P10). Participants perceived HP to be valuable beyond single-patient health in providing family members with support to promote health and prevent diseases and disabilities that can keep them away from the hospital.

*“Yes, it’s very important. It’s not all about managing diseases but trying to get the patients and family to stay healthy and not frequent in hospitals with disabilities and diseases. So… is part of our practice in the public health in this country”.*

*P13*

Participants acknowledged that CVDs have multiple causes and risk factors, and it is well within their scope to evaluate, monitor, and provide advice on these risk factors to prevent relapse and complications due to these risk factors.

*“Yes, most patients that come here maybe post-stroke; we start by educating and monitoring certain biochemical processes in the system like cholesterol level and triglycerides because those are the risk factors that can lead to a second stroke, which is very dangerous. Advise most of the patients that there is a probability that having a second stroke is very possible”*.

*P6*

Some of the participants perceived HP to be very valuable in maintaining long-term health and not just for the management of a condition within a clinical setting.

*“To me, I think it’s important because it will help to improve the patient’s health in the long term, not only at the clinic but even after, and it would also delay the occurrence of a new disease”*.

*P10*

Despite participants accepting that HP is within their professional role, there is some ambiguity with what constitutes the scope and standards of HP for some components such as nutrition, smoking, sleep, and alcohol use that are perceived to be outside the traditional scope of physiotherapy practice.

*“But from my part, education is part of it, but it becomes difficult when you enter another field which is not yours. Diet and nutrition are separate, so it is not easy for somebody to enter somebody’s field. So, we just do what we can do to help the patient”*.

*P9*

#### 3.2.2. Theme 2: Current Practice of PLHP for pwCVDs

1. Areas of current practice

Some participants assessed CVD risk factors in practice. This was limited and took differing forms among participants. Most of the participants reported no specific or objective assessment tool for HP components apart from weight measurement and the calculation of BMI.

*“Absolutely, all patients that pass through the clinic we take their BMI of all the patients. We know the importance of obesity as a risk factor for cardiovascular diseases in the body”.*

*P4*

The majority of the participants used only therapeutic assessments and approaches in practice with no lifestyle screening, as highlighted by P7.

*“I follow the conventional way of assessing a patient as you start with the demographic data, past medical history and all of that. Once I go through that procedure. I established the diagnosis and then that’s how I get to a conclusion on what the patient is suffering from”.*

*P7*

Some participants acknowledged delivering exercise and nutritional/dietary advice for patients more than any other component in their practice, with some expressing frequent engagement and confidence in delivering weight management advice. This was common among physiotherapists practicing in either public or private settings and reiterated by those involved in training physiotherapists.

*“So, we advise patients a lot on exercises and on diets. Because if you see the world today, many patients end up becoming overweight or hypertensive at a very young age. So, to avoid that, we advise patients a lot on their diet and regular exercises”.*

*P1*

While current practice tends to focus primarily on exercise, nutritional/dietary, and weight management interventions, the content and quality of these interventions were difficult to ascertain as no specific or objective measures were mentioned. The majority of the participants acknowledged assuming a counselling role in their practice to address a range of health issues. This was observed across lifestyle components, medical risk factors, and stress-related concerns from participants in practice and providing training.

*“With blood pressure, for example, at any time the patient visits, the blood pressure is taken. If it’s too high compared to the last time, then we need to sit the patient down and talk, what is happening? Why has your blood pressure gone up? What changed from the last time? How has your diet been? With all of that we can understand how to better manage the patient”.*

*P1*

While participants in the private sector tend to adopt a routine approach, those in the public sector tend to assume a general counselling role across multiple components of HP.

*“But the area that sometimes I feel comfortable with patients when counselling them, especially a patient with severe pain, I know that what I’m doing is just one-third of what can be done to help the patient. So, sometimes, I educate patients on positions that aggravate and relieve their pains. Sometimes I educate them, but I do lay emphasis on nutrition”.*

*P11*

Participants acknowledged the role of other health experts and engaged in appropriate referrals when they perceived they were limited in delivering relevant HP interventions, such as for nutritional issues. The referral process and HP practice were not influenced by the participants’ qualifications, but rather by the availability of experts and the participants’ length of service.

*“If a patient asks me for information about nutrition, generally, I will give the patient basic knowledge. But when they want deep knowledge, I’ll send them to a nutritionist. That’s what I do; when the patients tell me, for instance, that I’m not sleeping, I will ask why. Are you stressed out? Are you eating very well? But I’ll send it to a nutritionist for checking”.*

*P10*

However, beyond referrals, there was no evidence of meaningful collaboration and synergy with other clinicians to maintain high-quality care in routine practice. Different healthcare providers have a role to play, as highlighted by P10.

*“Yes, …is supposed to be a holistic care. We all have a part to play, so it’s not one person. I’ll be promoting health with respect to aspects that might be related to physiotherapy, and nurses will have theirs with respect to hygiene and sanitation. The doctors have theirs with respect to medications and all of that. So, if we can put our heads and hands in gloves together, then we are going to help the patients better. It’s important that all medical personnel work together in every aspect of a particular disease, for example, diabetes and all of that, to get to give the best of care to the patients”.*

*P10*

Some physiotherapists mentioned the importance of stress and lack of sleep and their potential impact on health and behaviour. However, most of the participants acknowledged little to no delivery of HP interventions around sleep, alcohol use, and smoking. Most physiotherapists pay little attention to these areas, irrespective of their level of competence, as they perceive the majority of the patients not to be honest about these components in some cases (P5).

*“For example, the ones that are very stressed by the work, normally those persons are not sleeping. They don’t sleep, they sleep very late, and they get up very early and those habits have effects on their health and the ability to act well or to respond well. When you don’t sleep well, you are very, very sensitive to many things around you”.*

*P12*

*“… the answer is no because a lot of people will not be honest about that. They are not always open about alcohol consumption. They are not honest about smoking”*

*P5*

2. Delivery methods

Participants engaging in HP delivered it through different formats and expressed different reasons for adopting preferred methods in some cases. The majority delivered promotional and preventive interventions verbally due to the limited availability of working space and promotional materials. Where participants have an interest to explore alternative delivery methods, they may also be limited by the skills and resources required to implement these in routine practice.

*“I can say that the reason why I’m doing it always verbally is because I don’t have the time to just write it, and to print it on paper, to help others. We know that not everybody likes to read. I can say that my brothers and sisters copied those habits, and they don’t read. I’m not saying that is the reason why I’m not doing it”.*

*P12*

*“I talk individually because first of all, I don’t have space to keep them to talk in group”.*

*P6*

Despite the dominant use of a one-to-one approach, a few (3/16) of the participants were aware of the benefits of group discussion and approaches to addressing HP issues. Sharing lived experiences during group discussions by patients was noted as a potential source of knowledge and motivation for other patients.

*“The truth is that when you do it in a group, it has more effect than when you do it individually because in a group, people can share their experiences, and then it helps them to really change. When you do it individually, the person might listen. But at the end of the day, they don’t have the courage to follow up the advice”.*

*P15*

3. Training needs

Participants expressed significant gaps in basic knowledge and concepts around HP, which made it difficult to deliver effectively.

*“Now, I know that I also have to learn more and do better as far as health promotion is concerned because I never thought of it as something I really have to take seriously”.*

*P15*

*“Time is not a barrier, but I only use my basic knowledge to educate patients. I do not have any document or support that I can use”.*

*P16*

While knowledge is not enough to drive behaviour change among patient groups, participants expressed and demonstrated a significant gap in using any behaviour change approaches or theories relevant to HP and physiotherapy practice. Participants could not reference any behaviour change strategy or model.

*“I am not aware of any specific cognitive or behavioural intervention, but I’ve implemented some behavioural changes. For instance, if you want to lose weight, don’t eat too many meals in a day; eat at a given time in a day. If you say you eat 2 times a day, don’t eat between those 2 times, don’t eat too late. At night, if you know you’re about to sleep at 9 or 10 pm, try to have your last meal around 6:00 pm; you make that persistent. But now, it is about a particular cognitive behavioural pattern. I don’t yet know about that”.*

*P14*

Also, participants demonstrated a substantial gap between objective assessment and monitoring of lifestyle factors in practice, with no objective or outcome measure tool mentioned. It is difficult to change an outcome if it is not well assessed and understood.

*“For lifestyle, just asking about their usual habits is the main way for me to assess it. And also, for behaviour change, no, I don’t really assess the behavioural change. I don’t have the skills to assess that”.*

*P3*

#### 3.2.3. Theme 3: Confidence in Delivering HP for pwCVDs

1. Level of confidence

Some participants, principally those engaged in training and teaching, expressed a good level of confidence in limited components such as exercise, physical activity, and stress management.

*“I am more confident, mostly in physical activity. Yes, stress management that’s counselling. I try to do counselling as much as possible”.*

*P2*

By contrast, some participants reported poor confidence in delivering across components of HP such as alcohol use, sleep, and smoking cessation, among others.

*“But the other aspects, I don’t feel so competent, so I tried to limit myself “.*

*P11*

2. Acquiring competence

The majority of the participants acknowledged gaining their knowledge and skills after formal physiotherapy training and through clinical exposure. Gaining more clinical experience was seen as the main way of building confidence and competence. Confidence in delivering advice was more associated with work experience than academic qualifications.

*“Yes, I think that came with the experience after so many years of dealing with people with these different conditions. You end up educating yourself or taking a course, and you improve these aspects because there are things you meet every day”.*

*P6*

Some participants gained confidence through reading and watching online content on HP. In some services, seminars and workshops have been sources of knowledge to improve participants’ competence. However, these workshops and seminars are not offered regularly at either institutional or national levels. Some participants acquired the necessary knowledge on HP principally from books and published articles.

*“I walk a lot with this Physio-works, Physiopedia and some online physiotherapy groups and so most information we are getting is usually from there online”.*

*P6*

*“Yes, ideally, we used to organize scientific meetings with presentations, but each department presents only once a year. So, when others are presenting, you learn as well when you’re presenting, they learn as well from you”.*

*P15*

*“Concerning diet, I had a book here called revolution des etudes du docteursArcaves an American. I used to explain to patients how to manage their weight, and at times, I gave them my own personal experience because formerly, I was a diabetic patient with the diet I had. Now I’m no more taking diabetic drugs”.*

*P9*

3. Challenges

Workload and limited time were intertwined factors that affected participants’ ability to provide HP intervention for both individual and group approaches. This was further influenced by the number of physiotherapists in the services and patient availability for such an education.

*“… sometimes that time to really sit and interact with the patient and the family, sometimes it’s difficult, but sometimes we just prioritize the treatment of the patient”*.

*P11*

Some participants (9/16) think that workload and time are not the major challenge to HP by comparison with the lack of necessary skills to deliver effectively on HP components.

*“Time is not a barrier, but I only use my basic knowledge to educate patients. I do not have any document or support that I can use”.*

*P16*

Participants acknowledged limited training opportunities from entry-level training and institutions, and limited continuous professional development courses around HP.

*“As a physiotherapist, I will not say that it’s has been really too much part of my formation or my training”.*

*P5*

*“Yes, the national society does contribute. The problem is that (HP training and courses) happens rarely. It can be like once annually, mostly towards World Physiotherapy Day when you have celebrations. Yes, but to say let’s plant something it’s very rare”.*

*P1*

HP is further challenged by the lack of resources, including local and national guidelines, limited infrastructure, such as working space for physiotherapy services and human resources such as physiotherapists employed in each service. Limited resources are available for infectious diseases, not NCDs in general.

*“Yes, I think we have an SOP that guides you in educating patients on some pathologies. SOPs, for example, are anything else apart from cardiovascular diseases. We have SOPs on how to counsel people with TB, HIV and all that on health promotion and other aspects of their lives. There are different pathologists, but for cardiovascular disease, specifically, I don’t think I found one, but for these diseases that are under programs in the country, they have SOPs”.*

*P13*

Participants reported that patients’ views, values, and perceptions sometimes challenge effective delivery of HP. Patients that are open, interactive, and value the advice can facilitate the process of HP.

*“Now, some prefer their gender; if it is a man, the man will prefer to talk to a man. If it is a woman, the woman we prefer to talk to the woman. If it’s a mother, I think that they don’t care if it is a man or a woman. When they are very aged, they don’t care about the gender of the therapist they just pull out”*.

P11

Participants reported challenges dealing with patients, but this could also be associated with the lack of skills to explore specific behaviours in a manner that is less invasive of privacy but elicits and strengthens motivation.

*“I don’t know whether it’s a cultural thing, but I look at it as it’s not really my field, and it’s kind of private when they open up, I’m ready. But if they do not open up, I don’t poke. Yes!”.*

*P5*

*“The patients have to trust you, before they can open up. I don’t think I see that, and I can confirm that many of them, I can say all of them are not open”.*

*P10*

## 4. Discussion

This is the first comprehensive qualitative study to explore the current practice, confidence, training needs, and perceptions of physiotherapists practising in Cameroon regarding their role in HP for pwCVDs. Physiotherapists believe that it is within their professional role to deliver HP interventions to pwCVDs in practice. Despite strong interest in HP, reported implementation of it among physiotherapists appears to be limited by challenges associated with education, resources, and confidence.

### 4.1. Perceptions of Physiotherapists’ Role in HP

Participants perceived HP to be within their professional roles and scope of practice. This aligns with our previous survey on HP practices, where respondents reported implementing several HP components [44]. The HP advice tends to focus on exercise, physical activity, and nutrition. Other components, such as smoking, sleep, and alcohol, were perceived to be outside the traditional scope of physiotherapy practice. These findings are consistent with prior research among physiotherapists in the UK and Nigeria, where HP practice focused on components of HP practice relating to exercise and physical activity [35,55]. While exercise and physical activity are the very fabric of the physiotherapy profession, perceptions towards other components are diverse and may be driven by contextual and prevalent risk factors. In addition to physical activity, in the UK, Walkeden and colleagues found that their participants were more likely to promote smoking cessation but less likely to provide advice on alcohol use, weight loss, or diet [35,44]. Beyond exercise and physical activity, our participants provide dietary advice, which may not be observed in a Western context with adequate dieticians and nutritionists [56]. The provision of dietary advice in this study aligns with comparable studies from Africa. These differences could be due to context-specific realities and further defined by what physiotherapists perceive to pose the greatest health risks. Limited attention to other risk factors such as sleep, alcohol use, and smoking cessation may be attributed to a poor understanding of the risk profile and the non-existence of experts in these areas in the Cameroonian context. There are high-profile calls for physiotherapy practice to be health-focused and for the scope to be broadened beyond exercise/physical activity to tackle the increasing NCD epidemic [28]. There is still ambivalence to embracing these calls by the physiotherapists in our study due to a lack of the knowledge and understanding of other components— such as smoking, sleep, and alcohol—required to engage in these components effectively. Secondly, some physiotherapists feel that wider HP is not within their scope of practice.

### 4.2. Current Practice of PLHP for pwCVDs

This qualitative study followed a scoping review, revealing that globally, physiotherapists engage in various HP activities, focusing on exercise/physical activity, diet, stress management, smoking, sleep, and alcohol intake, and using diverse strategies [57]. This aligns with the broader literature showing physiotherapists providing HP advice beyond traditional exercise and physical activity [35,55,56]. In the present study, participants reported engaging in various HP activities, notably exercise/physical activity and dietary interventions. This is consistent with our previous survey in Cameroon [44]. However, our qualitative data showed inconsistencies in the approaches and interventions across different behaviours, similar to findings from the UK and Germany [35,58]. This was associated with the lack of a consistent framework, clear structure, or specific approaches and content to deliver HP. Conversely, Walkeden and colleagues reported higher engagement and consistency among older physiotherapists on HP [35]. This may be associated with long-term clinical exposure, which makes them more confident to address behaviours. Consistency among older physiotherapists suggests confidence may be better developed with clinical exposures. Despite the availability of resources, training, and specific competencies related to HP, it is necessary to consider approaches to build confidence among young physiotherapists and enhance their engagement in multiple components of HP in practice.

In this study, participants showed limited awareness and use of behaviour change techniques across all HP components. They mostly provided general counselling without employing systematic approaches or specific techniques, such as goal setting, motivational interviewing, or the 5A (assess, advise, agree, assist, arrange) framework commonly used in primary care [38]. This is consistent with the findings from Germany, where Eisele and colleagues identified only 17 behaviour change techniques (BCTs) out of the 93 in the BCT taxonomy version one (BCTTv1) [58]. In contrast, only five BCTs were identified from our qualitative data. This may be due to the absence of guidelines and recommendations to support behaviour change intervention in the Cameroonian context. Globally, physiotherapists use a limited number of behaviour change techniques, possibly due to a lack of knowledge, experience, and perceptions of effectiveness [59].

Contrary to our quantitative data [44], qualitative data revealed a severe lack of resources and training opportunities for PLHP in Cameroon. Participants also reported difficulties managing uncooperative or unmotivated patients, similar to challenges reported in Germany and the UK [35,58]. This has important clinical and research implications, as the best methods for engaging and motivating patients, particularly those with multiple conditions, remain unclear. The effective use of BCTs and relevant resources are crucial, as prescription alone is insufficient to change behaviour in chronic conditions [60]. Given the ineffectiveness of therapeutic approaches for lifestyle conditions, relevant knowledge and skills are necessary when dealing with lifestyle conditions [60]. It is, therefore, necessary to promote the value of HP and the approaches that support better outcomes, such as BCTs, as there is no evidence that it is easily understood and implemented by physiotherapists in daily practice. Entry-level training programmes should incorporate HP and the evidence-based approaches that best support effective implementation.

### 4.3. Confidence in Delivering HP for pwCVDs

We report moderate confidence among the majority of participants, with some participants acknowledging the lack of confidence to deliver PLHP for pwCVDs either generally or on diet, sleep, alcohol, smoking, and stress management. Our previous survey showed similar results, but the current study highlights training gaps in HP [44]. This aligns with findings from Nigeria, where only 20% of respondents felt confident and adequately equipped with HP skills [55]. The low percentages demonstrate that most physiotherapists gained confidence and competence during clinical practice rather than during entry-level training. Likewise, McLean and colleagues reported that healthcare students in England gained more confidence in areas with better training and clear referral processes [61].

It is only in the last decade that physiotherapy courses have employed public health interventions beyond exercise and physical activity, such as nutrition, during physiotherapy training [62]. Findings from Ireland [63] demonstrate that less than a third of physiotherapists had some form of formal training on promoting health through nutritional advice. It is worth noting that nutrition intervention and other behaviours are still gaining global acceptance for the scope of physiotherapy practice [29,56].

Our participants used diverse methods and approaches, including the use of books, publications, workshops, seminars, and the Internet, with the use of social media in some cases to improve their competence and confidence to deliver HP. While diverse methods are welcome for different reasons in practice, the lack of standardization and competencies in HP in the training curriculum, coupled with the absence of continuous professional development opportunities on HP in Cameroon, is concerning. There is a need to invest in standardization and improved training opportunities, both during entry-level training and clinical practice.

### 4.4. Acquiring Competence in HP Practice

Our participants identified significant HP training gaps. These include poor awareness of behaviour change approaches, cognitive behavioural models, and frameworks such as the trans-theoretical model of readiness to change health behaviour; of motivational interviewing; of decision balance analysis; and of the 5 As endorsed by the WHO for individuals showing readiness to change. They also lacked basic knowledge of lifestyle components, such as healthy nutrition and alcohol intake, and did not utilize health assessment tools, including both general and condition-specific tools, and judgements of quality of life, life satisfaction and the health improvement card in practice. Without the awareness and appropriate tools to deal with behaviour and lifestyle issues, it remains a matter of chance for physiotherapists to be objectively successful in HP. Despite the majority of our participants being compassionate about HP for pwCVDs, it is unknown how effective these HP interventions are in Cameroon and to what extent patients can transfer these habits beyond their acute physiotherapy sessions into their everyday lives [64]. Similar gaps in formal HP training have been reported in Nigeria, where fewer than one-fifth of physiotherapists received training [55], and in Ireland, where many lacked training in areas like nutrition promotion [63]. Secondly, none of the participants reported using behaviour change models to support their counselling, which is concerning given the consensus that information alone is insufficient for behaviour change without intrinsic motivation [29,64]. Thirdly, participants were unaware of relevant assessment and outcome measurement tools, contributing to inconsistent practices and poor communication between physiotherapists and other clinicians. Addressing these gaps theoretically and practically may improve the capacity to deliver HP with confidence and align with the global call to prioritize health-based competencies in practice and entry-level education to effect multiple health behaviour changes to tackle the epidemic of NCDs [29]. Currently, there is no benchmark study on the content of the contemporary entry-level curriculum for physiotherapy training in Cameroon, either generally or based on content, training, and skill competence for HP. Continuous professional education and institutional and self-learning opportunities have been ways to improve HP knowledge and skills; however, these opportunities are irregular. While continuous professional education offers some improvement, regular and formal training based on global principles, adapted to local needs through evidence-based approaches, is essential for improving HP outcomes [29].

Participants highlighted the lack of equipment and working alone as significant challenges. Consistent with prior findings in Nigeria, human and HP-related resources were identified as major barriers [33]. By contrast, a study in Germany by Eisele and colleagues identified patient-related factors, such as a lack of enthusiasm for exercise and preference for passive treatments, as the primary barriers. These differences could be due to the availability of human resources and infrastructures in Germany. This underscores the global call to expand the rehabilitation workforce, particularly in Africa, where the demand is seen as critical [65].

### 4.5. The Implications of the Findings

There is a need for greater emphasis on public health and HP in the undergraduate physiotherapy curriculum. Coupled with a strong understanding of behaviour change theories and techniques, this would better equip graduating physiotherapists to effectively address lifestyle-related conditions within the Cameroonian context [29].

Relevant stakeholders including CASP, the Ministry of Public Health, non-governmental organizations, and policy makers should invest in evidence-based training opportunities for physiotherapists. CASP should develop and implement robust, continual professional education opportunities in public health and HP for qualified physiotherapists, leading to defined competencies with certification [29]. CASP should collaborate with the Ministry of Public Health to train and disseminate community health workers on NCD prevention strategies. These community health workers may be able to disseminate information through local structures that may be more acceptable and accessible to the populace and in a language that can easily be understood. Expert patient groups and leaders are viable ways to facilitate the dissemination of public health messages on a large scale [66].

Moreover, physiotherapists in Cameroon must enhance collaboration with other healthcare professionals beyond patient referrals. While knowing when to refer patients is essential, it is equally important to establish communication pathways and systems that facilitate patient care and improve specific health outcomes [29].

### 4.6. Strengths and Limitations

Participants were drawn from over 10 collaborating health services across four regions of Cameroon, which enhanced the transferability and credibility of these findings. Aligned with a constructivist methodology, it is recognized that the researchers’ preconceived ideas may have influenced the inquiry’s design. To minimize bias, iterative reflexivity was utilized to acknowledge the primary researcher’s role in shaping the data and analysis [67]. Data saturation was achieved before interviews were terminated, indicating that all salient concepts were likely identified.

### 4.7. Recommendation for Future Studies

Future studies should employ different designs to better explore HP practice and capacities to address specific health behaviours among physiotherapists in Cameroon. Intrapersonal factors such as duration in the profession, qualifications, and income status should be explored in future studies. Other factors, such as working context and cultural and religious factors, should also be studied to ascertain their influence on HP practice. The findings of such studies will better inform future interventions and expansion in rural areas. Our findings suggest physiotherapists do refer patients to other health professionals, but further studies are warranted to establish how effective the referrals and collaborations are.

## 5. Conclusions

In this qualitative study of 16 Cameroonian physiotherapists, participants reported that a broad range of HP practices fall within their scope of practice. They felt comfortable recommending exercise, physical activity, and diet as key areas of HP under their purview. However, they felt less confident providing advice on smoking cessation, sleep hygiene, and alcohol use, among others. Despite existing interest and engagement in HP, the data revealed a lack of awareness and application of behaviour change strategies, limited understanding of core HP components, and an absence of objective assessment and monitoring tools for various conditions in clinical practice.

To better address the growing challenges of lifestyle-related conditions in Cameroon, physiotherapists must be better equipped to deliver HP effectively in clinical practice. This requires investment in creating a culture of HP that emphasizes evidence-based interventions. Stakeholders such as CASP and the Ministry of Public Health should organize mandatory training to improve competence and confidence in delivering all aspects of HP, ensuring standardization and the use of objective measures in clinical practice and beyond.

## Figures and Tables

**Table 1:** Characteristics of physiotherapists who participated in qualitative interviews (n = 16).

Sn.	Age	Qualification	Employment Type	Working Experience	Sector
P1	30	MSc.	Clinician/Academic	4 years	Private
P2	34	MSc.	Clinician	6 years	Public
P3	40	BSc.	Clinician/Academic	13 years	Private
P4	61	BSc.	Clinician/Academic	33 years	Retired (Public/Private)
P5	46	BSc.	Clinician	17 years	Mission (Catholic Facility)
P6	37	BSc.	clinician	4 years	Private
P7	36	BSc.	Clinician	8 years	Public
P8	42	HND	Clinician	10 years	Private
P9	63	HND	Clinician	36 years	Retired (Public/Private)
P10	33	HND	Clinician	9 years	Private
P11	38	BSc.	Clinician	13 years	Public
P12	32	BSc.	Clinician	4 years	Private
P13	43	MSc.	Clinician/Academic	12 years	Public/Private
P14	33	MSc.	Clinician/Academic	6 years	Private
P15	40	BSc.	Clinician	10 years	Public
P16	38	HND	Clinician	11 years	Public/Private

P: Participants, HND: Higher National Diploma, BSc.: Bachelor of Science, MSc.: Master of Science.

**Table 2:** Higher- and lower-order themes on PLHP practice and perceptions among Cameroonian physiotherapists (n = 16).

Dimensions	Higher-Order Themes	Lower-Order Themes
Perceptions of their roles in HP	The professional role of PTs	Scope of practice
		Prevention of diseases and disabilities
The current practice of PLHP	Areas of current practice	Assessing lifestyle
		Exercise and diet
		General advice and counselling
		Referrals and multidisciplinary collaboration
		Smoking, sleep, and alcohol
	Delivery methods	Verbal discussion
		Group education or exercise
		Written or printed out
	Training needs	Behaviour change approaches
		Basics on health education
		Assessing lifestyle behaviour
Confidence in delivering HP for pwCVDs	Level of confidence	Perceived as moderate
		Perceived as low
	Acquiring competence	During training and clinical experience
		Internet
		Seminars and workshops
		Books and publications
	Challenges	Workload and time
		Training
		Lack of resources
		Perceptions of patients

## Data Availability

The original contributions presented in this study are included in the article/Supplementary Materials. Further inquiries can be directed to the corresponding author.

## Supplementary Materials

The following supporting information can be downloaded at https://www.mdpi.com/article/10.3390/healthcare13101172/s1, File S1: Topic Guide; Table S1: Final themes and categories on the PLHP practice and perceptions among Cameroonian physiotherapists with example reference.
