# Supplementary material for: Cameroonian Physiotherapists’ Practice, Confidence, and Perception of Health Promotion for People at Risk or with Cardiovascular Diseases: A Qualitative Study
Source: Healthcare (Basel). 2025 May 17;13(10):1172. doi: 10.3390/healthcare13101172 (PMC12110929; doi:10.3390/healthcare13101172)
Supplement: Supplementary file 1 [file healthcare-13-01172-s001.zip › healthcare-3547626-supplementary.pdf]

Supplementary file Table S1: Final themes and categories on the PLHP practice and perceptions among Cameroonian physiotherapists with example reference (quotes) (n=16)

**Dimensions**	**Higher order themes**	**Lower order themes**	**files**	**References**	**Sampled reference (quotes)**
Perceptions of PT role in HP	Professional role of PTs	Scope of practice	11	20	*"It is important because the health promotion goes with the physiotherapy. You offer it because it's going to help people get more from what they are already getting. And especially in the private practice, you need them to come back. You need them to refer people. So, you need that your service is better than the regular service anywhere else"* P6<br>*"But from my part, education is part of, but it becomes difficult when you enter another field which is not yours. Diet and nutrition are separate, so it is not easy for somebody, someone, to enter somebody's field. So, we just do what we can do to help your patient"* P9.
		Prevention of diseases and disabilities	14	37	*"Yes, it's very important. It's not all about managing diseases but trying to get the patients and family to stay healthy and not frequent in the hospital with disabilities and diseases. So, it's always practiced, I think, is part of our practice in the public health in this country"* P13<br>*"Yes, most patients that come here maybe post-stroke; we start by educating and monitoring certain biochemical processes in the system like cholesterol level and triglycerides because those are the risk factors that can lead to a second stroke, which is very dangerous. Advise most of the patients that there is a probability that having the second stroke is very possible".* P6<br>*"To me I think it's important because it will help to improve the health of the patient in the long term, not only at the clinic, but even after and also it would delay the occurrence of a new disease"* P10.
Current practice of PLHP	Areas of current practice	Assessing lifestyle	12	27	*"I follow the conventional way of assessing a patient as you start with the demographic data, past medical history and all of that. Once I go through that procedure. I established the diagnosis. Yes, that's how I do that. I follow the conventional way of assessing the patient, and then that's how I get to a conclusion on what the patient is suffering from", P7.*<br>*"Absolutely, all patients that pass through the clinic we take their BMI of all the patients. We know the importance of obesity as a risk factor for cardiovascular diseases, even neurology or many other systems in the body" P4.*
		Exercise and diet	14	37	*"So, we advise patients a lot on exercises and on diets. Because if you see the world today, many patients end up becoming overweight or hypertensive at a very young age. So, to avoid that, we advise patients a lot on their diet and regular exercises" P1.*<br>*"I think here at our health institute, we mostly talk about exercises, we also talk on diet because certain diet, we try to explain to the patients the importance of diet to be nourished*

					and to avoid certain complications" P2.
		General advice and counselling	13	29	*"With blood pressure, for example, at any time the patient visits, the blood pressure is taken. If it's too high compared to the last time, then we need to sit the patient down and talk, what is happening? Why has your blood pressure gone up? What changed from the last time? How has your diet been? With all of that we can understand how to better manage the patient" P1.*<br>*"But the area that sometimes I feel comfortable with patients is mostly when counseling them, especially a patient with severe pain, I know that what I'm doing is just one-third of what can be done to help the patient. So sometimes I educate patients on the positions that aggravate and relieve their pains. Sometimes I educate them, but I do lay emphasis on nutrition"* P11.
		Referrals and multidisciplinary collaboration	7	11	*"If patient ask me, information about nutrition, generally I will give to the patient basic knowledge. But when they want deep knowledge, I'll send them to a nutritionist. That's what I do, when the patients tell me, for instance, that I'm not sleeping, I will ask why? Are you stressed up, are you eating very well? But I'll send it to a nutritionist for checking",* P10.<br>*"Yes, health care naturally, I don't know how to put it is supposed to be a holistic care. We all have a part to play, so it's not one person. I'll be giving health promotion with respect to aspects that might be related to physiotherapy and nurses will have theirs with respect to hygiene and sanitation. The doctors have theirs with respect to medications and all of that. So, all together if we put our heads, hand in gloves, then we are going to help the patients better. It's important that all medical personnel work together in every aspect of a particular disease, for example, diabetes and all of that, to get to give the best of care to the patients"* P10.
		Smoking, sleep and alcohol	13	22	*"For example, the one that are very stressed by the work, normally those persons are not sleeping. They don't sleep, they sleep very late, and they get up very early and those habits have effects on their health and the ability to act well or to respond well. When you don't sleep well, you are very, very sensitive to many things around you", P12.*<br>*"I think the first, no, the answer is no because a lot of people will not be honest about that. They are not always about the alcohol consumption. They are not honest about smoking", P5*
	Challenges	Workload and time	8	23	*"There are barriers among which are sometimes you get so pressured that you just want to administer the treatment, call in the next patient, sometimes that time to really sit and interact with the patient and the family, sometimes it's difficult, but sometimes we just prioritize the treatment of the patient"* P11<br>*"Time is a barrier because most of them, since I deal with handicapped people, they may have a driver. The car can be a taxi and when the taxi comes, the driver will not wait. I don't have opportunity to move from house to house, because it's not really my job",* P9.

		Education and Training	9	26	*"As a physiotherapist, I will not say that it's has been really too much part of my formation or my training" P5.* *"Yes, the national society does contribute. And the problem is it happens rarely. It can be like once annually mostly towards world physiotherapy day when you have celebrations. Yes, but to say let's plant something it's very rare", P1*
		Lack of resources	12	37	*"Yes, I think we have SOP that guides you to educate patients in some pathologies. SOPs for example any other thing apart from cardiovascular diseases. We have SOPs on how to cancel people with TB, HIV and all that on health promotion and other aspects of their lives. There are for different pathologists, but for cardiovascular disease, specifically, I don't think I found one, but for these diseases that are under programs in the country, they have SOPs, standard SOPs", P13.*
		perceptions of patients	13	35	*"Now, some prefer their gender, if it is a man, the man, will prefer to talk to a man. If it is a woman, the woman we prefer to talk to the woman. If it's a mother, I think that they don't care if it is a man or a woman. When they are very aged, they don't care about the gender of the therapist they just pull out."* P11 *"I don't know whether it's a cultural thing, but I look at it as it's not really my field, and it's kind of private when they open, I'm ready. But if they do not open, I don't poke. Yes!"* P5.
	Training needs	Behavior change approaches	11	27	*"I am not aware of any specific, cognitive or behavioral intervention, but I've implemented some behavioral changes. For instance, if you want to reduce weight, don't eat too many meals in a day, eat at a given time in a day. If you say you eat 2 times a day, don't eat in between those 2 times, don't eat too late. At night, if you know you're about to sleep at 9 or 10 pm, try to take your last meal around 6:00 pm, you make that persistent. But now about a particular cognitive behavioral pattern. I don't yet know about that".* P14
		Basics on health education	12	22	*"Now, I know that I also have to learn more and do better as far as health promotion is concerned, because I never thought of it as something I really have to take serious",* P15. *"Time is not a barrier, but I only use my basic knowledge to educate patients. I do not have any document or support that I can use",* P16.
		Assessing lifestyle behavior	13	36	*"For lifestyle just by asking their usual habits is the main way for me to assess it. And, for behavior change, no, I don't really assess the behavioral change. I don't have the skills to assess that".* P3.
Competence in delivering HP for pwCVDs	Level of competence	Perceived as moderate	10	16	*"I am more confident, mostly in physical activity. Yes, stress management that's counseling. I try to do counseling as much as possible". P2* *"I feel confident and competent because I'm a physiotherapist course instructor, teaching physiotherapy in cardiovascular and respiratory systems. By doing that, we explored a lot of documents, textbooks and so with what, I have acquired and what I share with the student, it gives me some leverage to be able to transmit that to my patient or to other individuals", P14.*

		Perceived as low	9	12	*"But the other aspects, I don't feel so competent, so I tried to limit myself "*P11
	Acquiring competence	During training and clinical experience	14	20	*"Yes, I think that came around with the experience after so many years of dealing with people with these different conditions. You end up educating either yourself or taking a course, and you improve these aspects because there are things you meet every day" P6.*
		Internet	6	6	*No, we Google soft copy most of the time we go directly online and get the Information P4*<br>*I walk a lot with this Physio-works,Physiopedia and some online physiotherapy groups and so most information we are getting is usually from there online, P6.*
		Seminars and workshops	4	6	*"Yes, ideally, we used to organize scientific meetings with presentations, but each department presents only once a year. So, when others are presenting, you learn as well when you're presenting, they learn as well from you" P15*
		Books and publications	4	6	*"Yeah, health education, mostly materials, like studies that have been carried out and you get them from books, publications. I'm so confident that if I have to apply a material that I have seen online, and I'm convinced that it works"* P11<br>*"Concerning diet, I had a book here called revolution des etudes du docteursArcaves an American. I used to explain to patient how to manage their weight and at times I give them my own personal experience, because formerly I was a diabetic patient with the diet I had. Now I'm no more taking diabetic drugs"* P9.
	Delivery methods	Verbal discussion	7	9	*"I talk individually because first of all, I don't have space to keep them to talk in group", P6,15,16*<br>*"It's just one on one. Yes, I know that group discussion exists, but in that case, many patients are not very open. The patients have to trust you, before they can open up. I don't think I see that, and I can confirm that the many of them, I can say all of them are not open" P10.*
		Group education or exercise	5	8	*"The truth is that when you do it in a group, it has more effect than when you do it individually, because in group people can share their experiences, and then it helped them to really change. When you do it individually, the person might listen. But at the end of the day, they don't have the courage to follow up the advice" P15*
		Written or print out	2	3	"*I can say that the reason why I'm doing it always verbally is because I don't have the time to just write it, and to print it on papers, to help others. We know that it's not everybody that always likes to read. I can say that my brothers and sisters copied those habits and they don't read. I'm not saying that is the reason why I'm not doing it" P12*
