# Supplementary material for: Cameroonian Physiotherapists’ Practice, Confidence, and Perception of Health Promotion for People at Risk or with Cardiovascular Diseases: A Qualitative Study
Source: Healthcare (Basel). 2025 May 17;13(10):1172. doi: 10.3390/healthcare13101172 (PMC12110929; doi:10.3390/healthcare13101172)
Supplement: Supplementary file 1 [file healthcare-13-01172-s001.zip › SUPPLEMENTARY FILE S1.pdf]

Supplementary File S1: TOPIC GUIDE

THANKS FOR ACCEPTING TO TAKE PART IN THIS STUDY

1. Can I start by asking you what health promotion means to you as a physiotherapist?
    a. Your understanding of the term health promotion
    b. In relation to practice
2. Do you practice health promotion to enable patients to improve or increase control over their condition or risk factors that can lead to CVD?
    a. Frequently or rarely?
    b. specific components of health promotion?
3. Please tell me more about which areas of health promotion in practice you feel comfortable engaging your patients?
    a. Exercise and physical activity
    b. Diet/nutrition
    c. Alcohol, smoking and sleep counselling?
4. Why is health promotion practice important to you?
    a. Prevention of diseases (primary, secondary and tertiary)
    b. Improve clinical outcomes
5. What importance does health promotion hold in your practice?
    a. Valuable component of your practice?
6. Do you generally assist patients with strategies to adopt and maintain new habits? If yes, what are some of the habits and strategies?
    a. Which components?
7. Where you work, are there any barriers which make health promotion difficult?
    - Individual (knowledge, training, time …)
    - Environmental (resources, space…)
    - Institutional (guidelines, collaborators, mentors...)
    - Systematic (National and international guidelines…)
8. How competent are you in delivering health promotion activities for pwCVDs?
    a. Understanding of what to do in each case based on your training/knowledge
9. To what extent do you feel competent at delivering health promotion activities for pwCVDs?
    a. Confidence
10. How did you gain such competence?
    a. During training
    b. Clinical placement
    c. CPDs
11. Are you aware of cognitive behavioral interventions, strategies, and therapists? If yes, please tell me more about how you employ that into health promotion practice.
    a. Theory to aid in behavior change
    b. Specific strategies
12. Are you comfortable assessing lifestyle and behavior changes for a patient? If so, can you give me some practical examples of doing this?
    a. Specific outcomes measures
    b. What you do in routine practice
13. Do you generally discuss challenges patients may face while trying to improve in any areas? If so, can you give me some practical examples of doing this?
    a. Resources

    b. Providing best evidence and options

14. Are there any theories or concepts you employ when guiding you to assess your patient's willingness to engage in proposed changes?
    a. Adopted pattern for routine practice
    b. Specific techniques
15. Do you generally assist your patient to know the optimal recommended values or best practice guide for any area of intervention you propose? Which sources do you use for this information?
    a. Physical activity
    b. Sleep
    c. Alcohol
    d. Weight
16. Any questions or last word for me?

BEFORE WE FINISH, IS THERE ANYTHING YOU WOULD LIKE TO ADD

WE'LL FINISH HERE – THANKS FOR TAKING PART
